# Human α-Defensin-6 Neutralizes *Clostridioides difficile* Toxins TcdA and TcdB by Direct Binding

**DOI:** 10.3390/ijms23094509

**Published:** 2022-04-19

**Authors:** Lara Barthold, Sebastian Heber, Christoph Q. Schmidt, Marion Gradl, Gilbert Weidinger, Holger Barth, Stephan Fischer

**Affiliations:** 1 Institute of Pharmacology and Toxicology, Ulm University Medical Center, 89081 Ulm, Germany; lara.barthold@uni-ulm.de (L.B.); sebastian.heber@uni-ulm.de (S.H.); 2 Institute of Pharmacology of Natural Products and Clinical Pharmacology, Ulm University Medical Center, 89081 Ulm, Germany; christoph.schmidt@uni-ulm.de; 3 Institute of Biochemistry and Molecular Biology, Ulm University, 89081 Ulm, Germany; marion.gradl@uni-ulm.de (M.G.); gilbert.weidinger@uni-ulm.de (G.W.)

**Keywords:** *Clostridioides difficile* infections, human antimicrobial peptide, α-defensin-6, bacterial AB-type protein toxins, large clostridial glucosylating toxins TcdA and TcdB, toxin inhibitor, zebrafish

## Abstract

Rising incidences and mortalities have drawn attention to *Clostridioides difficile* infections (CDIs) in recent years. The main virulence factors of this bacterium are the exotoxins TcdA and TcdB, which glucosylate Rho-GTPases and thereby inhibit Rho/actin-mediated processes in cells. This results in cell rounding, gut barrier disruption and characteristic clinical symptoms. So far, treatment of CDIs is limited and mainly restricted to some antibiotics, often leading to a vicious circle of antibiotic-induced disease recurrence. Here, we demonstrate the protective effect of the human antimicrobial peptide α-defensin-6 against TcdA, TcdB and the combination of both toxins in vitro and in vivo and unravel the underlying molecular mechanism. The defensin prevented toxin-mediated glucosylation of Rho-GTPases in cells and protected human cells, model epithelial barriers as well as zebrafish embryos from toxic effects. In vitro analyses revealed direct binding to TcdB in an SPR approach and the rapid formation of TcdB/α-defensin-6 complexes, as analyzed with fluorescent TcdB by time-lapse microscopy. In conclusion, the results imply that α-defensin-6 rapidly sequesters the toxin into complexes, which prevents its cytotoxic activity. These findings extend the understanding of how human peptides neutralize bacterial protein toxins and might be a starting point for the development of novel therapeutic options against CDIs.

## 1. Introduction

Healthcare-associated infections (HAIs) caused by pathogens such as bacteria, fungi or viruses arise in several healthcare contexts, e.g., in hospitals, long-term nursing facilities and outpatient settings. As much as 6.5% of all hospital patients in the European Union are affected by HAIs [1]. The most common organism in HAIs is *Clostridioides* (*C.*) *difficile*, formerly known as *Clostridium difficile* [2]. *C. difficile* is a Gram-positive, anaerobic, spore-forming bacterium of the human gastrointestinal tract. This important nosocomial pathogen can cause toxin-mediated *C. difficile* infections (CDIs) with clinical presentations ranging from asymptomatic infections to diarrhea, pseudomembranous, fulminant colitis and ultimately death [3]. No matter what clinical symptoms appear, the cause is always the production and the release of protein toxins. After germination in the gut, the bacterium produces proteinaceous exotoxins which act on intestinal epithelial cells, thereby leading to tissue injury and as a direct consequence to the development of clinical symptoms. To date, three different toxins are known to be produced by *C. difficile*: the two large glucosylating toxins A (TcdA) and B (TcdB) and the binary ADP-ribosyltransferase toxin CDT [4,5]. However, the presence of TcdA and TcdB is sufficient to fully develop the clinical pattern [6,7]. TcdA and TcdB share a sequence identity of about 50% and have a size of 308 and 270 kDa, respectively [8,9]. Both toxins consist of four functionally different domains: the N-terminally located, enzymatically active glucosyltransferase domain (GTD), an autoprotease or self-cutting domain (APD), a translocation domain responsible for pore-formation in endosomal vesicles and a binding domain which mediates the binding of the toxin to cell surface receptors. The latter also contains the C-terminally located repetitive oligopeptide sequences (CROPS), which are also involved in cell binding.

TcdA and TcdB enter and intoxicate their host target cells via a sophisticated, multistep pathway. After binding to cell surface receptors and receptor-mediated endocytosis, the toxins undergo conformational changes in acidified endosomes [10]. This enables the insertion and pore formation in endosomal membranes with subsequent translocation of the APD and GTD to the cytosolic side of the endosomes [11,12]. Here, inositol hexakisphosphate (InsP_6_) induces autoproteolytic cleavage by the APD and thus the release of the GTD into the cytosol [13,14]. Once in the cytosol, the GTD glucosylates GTPases of the Rho-/Ras-family located at the cell membrane, among which Cdc42, Rho and Rac1 represent the key toxin substrates [15,16]. On the cell level, mono-glucosylation and thereby the inactivation of these GTPases results in the secretion of defined cytokines, changes in the cytoskeleton structure accompanied with cell rounding and loss of cell–cell contacts, cell cycle arrest and cell death [17]. The in vivo inactivation of these molecular switches is thought to be the major reason for intestinal damage, gut barrier disruption and the occurrence of clinical symptoms such as diarrhea [18,19].

The benchmark for treating CDIs is still the use of some specific antibiotics such as vancomycin, metronidazole or fidaxomicin [20]. More and more attention is also being paid to the direct inactivation of the secreted toxins, for example by using anti-toxin-antibodies such as bezlotoxumab or actoxumab [21,22]. Following the approach of directly inactivating the secreted toxins and based on previous findings of our group and of others, we investigated the effects of human endogenous antimicrobial peptides on the *C. difficile* toxins TcdA and TcdB. Here, of particular interest is the group of defensins. Defensins are small peptides (2–5 kDa) and usually comprise six cysteines, with three intramolecular disulfide bonds stabilizing the overall architecture. As major effectors of the innate immune system, defensins feature an impressive variety of immune modulatory and antimicrobial activities [23]. Moreover, it became evident that specific defensins, especially α-defensins, neutralize some medically relevant bacterial protein toxins, including TcdB [24]. These findings were further extended by our group to reveal that α-defensin-1 and α-defensin-5 (α-def-5) also inactivate TcdA and CDT, in addition to TcdB [25,26]. However, α-defensin-6 (α-def-6) has not been studied in this context so far. α-def-6 is produced by Paneth cells in the small intestine and, in contrast to other α-defensins, displays negligible microbiocidal activity. It rather provides defense against pathogens by self-assembling into extended higher-order oligomers, which have been shown to entangle bacteria, thereby preventing them from invading the intestinal epithelium [27]. Prompted by these divergent characteristics, it was intriguing to examine α-def-6 also in the context of toxin inactivation.

In the present study, the protective effect of α-def-6 on the toxic effects of TcdA and TcdB was demonstrated in vitro in a set of cell-based approaches as well as in vivo in a zebrafish embryo model, and the underlying molecular mechanism was discovered.

## 2. Results

### 2.1. α-def-6 Directly Interacts with TcdB without Affecting Its Autoprotease or Enzyme Activity

**Figure 1:**
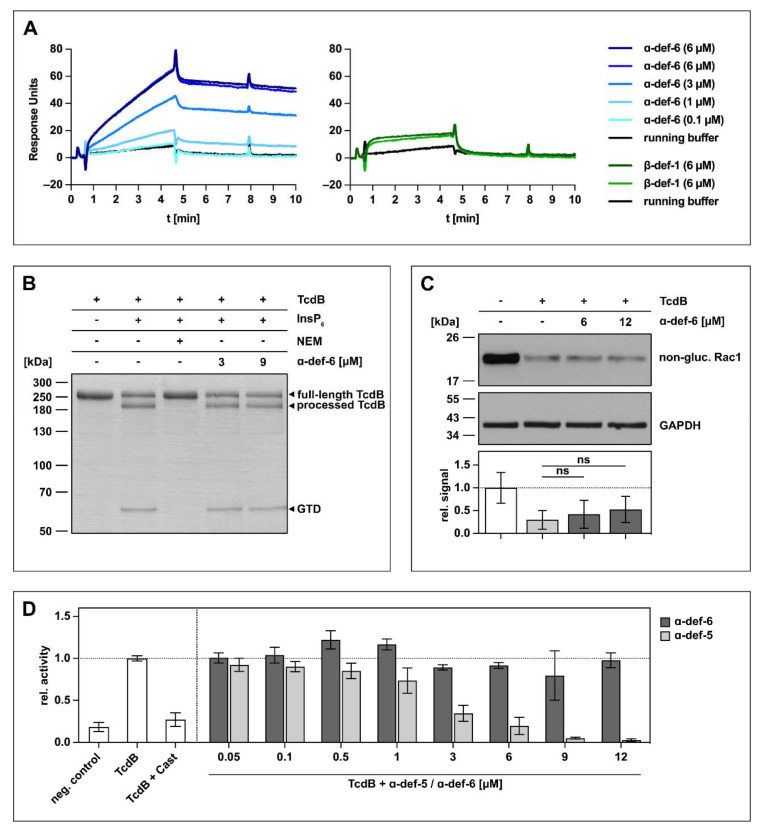
α-def-6 directly binds to TcdB in vitro but does not affect the autoproteolytic- and glucosyltransferase activity of TcdB. (**A**) 2400 RUs of biotinylated TcdB were immobilized via streptavidin-biotin binding onto a streptavidin sensor chip, and α-def-6 (concentrations as indicated) was applied to the chip for 4 min followed by a dissociation phase with a flow of running buffer. A solution of 6 µM of α-def-6 was injected twice to assess reproducibility, and β-def-1 (6 µM) served as a control. The flow rate was 25 µL/min. (**B**) The autoproteolytic activity of TcdB (2 µg) in the presence of InsP_6_ (1 mM) was evaluated in the absence or presence of α-def-6 (3 µM, 9 µM). *N*-ethylmaleimide (NEM; 1 mM), an inhibitor of the autoproteolytic activity of TcdB, was included as the control. After 1 h incubation at 37 °C, the samples were subjected to SDS-PAGE, and the protein was visualized by Coomassie staining. A representative gel is shown (*n* = 2). (**C**) CaCo-2 cell lysate (40 µg) as source of Rac1 was incubated with TcdB (50 ng) in the absence or presence of α-def-6 (6 µM, 12 µM) for 2 h at 37 °C. After SDS-PAGE and Western blotting, non-glucosylated Rac1 was detected with a specific antibody. GAPDH served as the loading control. A representative Western blot is shown. Relative signal intensities normalized to loading control are given as mean ± SD of four biological replicates, each with two technical duplicates (*n* = 4). Significance was tested with a one-way ANOVA combined with Dunnett’s multiple comparison test (ns = not significant *p* > 0.05). (**D**) UDP-Glo^TM^ glucosyltransferase assay was performed to analyze the glucosyltransferase activity of TcdB. TcdB (200 pM) was incubated with α-def-5 or α-def-6 (concentrations as indicated) in the presence of recombinant Rac1 (5 µM) as substrate. Castanospermine (Cast; 10 mM), a known inhibitor of the glucosyltransferase activity, was included as a control. Reactions were started with the addition of UDP-glucose (100 µM) and allowed to proceed for 1 h at 37 °C. Samples were combined with UDP detection reagent, and luminescence was measured. Values represent mean ± SD of at least three biological replicates, each with three technical replicates (*n* ≥ 3).

The initial experiments to investigate an interaction between α-def-6 and clostridial glucosylating toxins were carried out with TcdB. At first, the direct interaction was tested using surface plasmon resonance. To this end, biotinylated TcdB was immobilized onto a sensor chip, and different concentrations of α-def-6 (ranging from 0.1 to 6 µM) were applied in running buffer. As shown in Figure 1A (left panel), a direct, concentration-dependent interaction between α-def-6 and TcdB was detected. As it was reported earlier that β-defensins (specifically β-def-1) do not interact with bacterial toxins including TcdB, β-def-1 was used as control to exclude non-specific interactions between TcdB and defensins [24,25,26,28]. As expected, the interaction between immobilized TcdB and β-def-1 was much weaker in combination with a faster washout compared to the interaction between α-def-6 and TcdB in the same approach (Figure 1A, right panel).

Based on the finding that TcdB directly binds to α-def-6 in vitro, a set of experiments was performed to investigate the consequences of this interaction for TcdB. Although α-def-6 and TcdB interact directly, the release of the glucosyltransferase domain by the autoprotease domain in the presence of inositol hexakisphosphate (InsP_6_) was not affected (Figure 1B). Here, *N*-ethylmaleimide (NEM) was used as an established inhibitor of the autoprotease activity of TcdB and prevented the autocatalytic processing of TcdB under the chosen conditions [14]. Moreover, almost no inhibition of the enzymatic activity of the glucosyltransferase domain of TcdB was observed in vitro even at α-def-6 concentrations up to 12 µM (Figure 1C). Consistent with this finding, α-def-6 was also not able to inhibit glucosyltransferase activity of TcdB in an additional UDP-Glo^TM^ glucosyltransferase assay (Figure 1D). Here, castanospermine (Cast) was used as an established glucosyltransferase inhibitor [29]. Noteworthy, α-def-5, a structurally similar defensin, showed a clear concentration-dependent reduction of glucosyltransferase activity.

### 2.2. Interaction between α-def-6 and TcdB Leads to the Rapid Formation of Toxin-Inhibitor-Complexes

**Figure 2:**
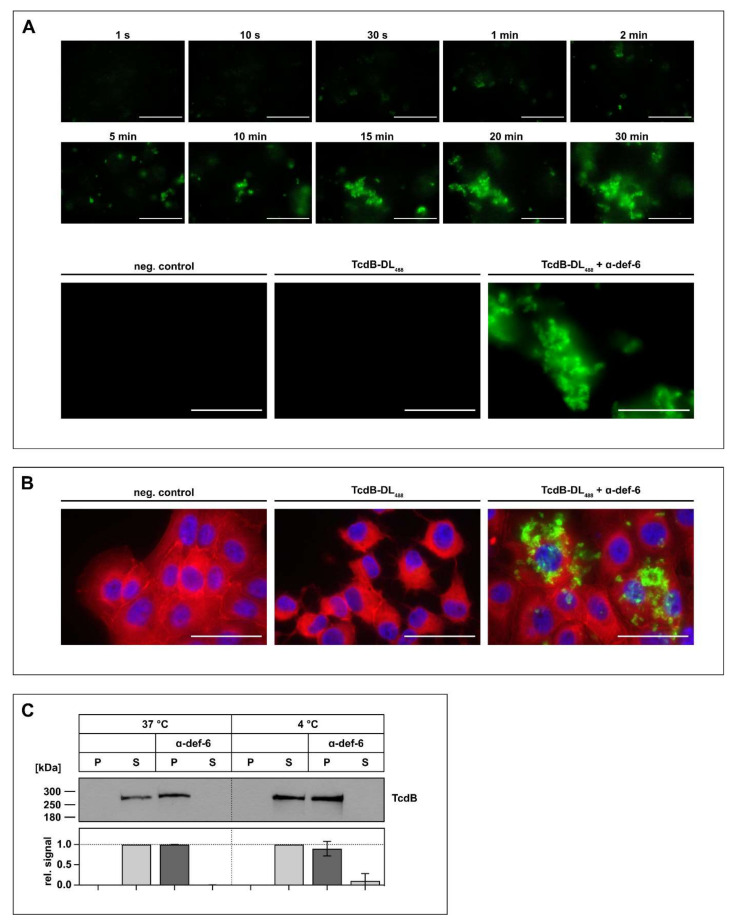
The incubation of α-def-6 and TcdB leads to rapid aggregation. (**A**) α-def-6 (6 µM) was added to TcdB-DL_488_ (30 nM) in PBS, and image acquisition with an epifluorescence microscope was started immediately. Aggregation was allowed to proceed for 0.5 h at 37 °C while images were being taken. Representative images of various time points are depicted to show the rapid progression of aggregation over time (upper panel). TcdB-DL_488_ (30 nM) alone in PBS was used as a positive control, while the same volume of Dylight488 treated PBS in PBS served as a negative control. Images after 0.5 h at 37 °C are depicted (lower panel), (*n* ≥ 2). (**B**) Vero cells were intoxicated with TcdB-DL_488_ (22 nM) in the absence or presence of α-def-6 (6 µM) for 0.5 h at 37 °C. Cells subjected to the same volume of DyLight488-treated PBS, as used TcdB-DL_488_, served as control. Cells were fixed and permeabilized. Nuclei (blue) were stained with Hoechst33342 and actin (red) with SiR-actin. Representative images taken with an epifluorescence microscope are depicted (*n* = 2). Scale bars correspond to 50 µm. (**C**) TcdB (50 ng) was incubated with or without α-def-6 (6 µM) in PBS for 0.5 h at 37 °C. Samples were centrifuged to segregate aggregates and divided into a supernatant (S) and pellet (P) fraction. Fractions were subjected to SDS-PAGE followed by Western blotting. TcdB was detected. Signals were analyzed as the ratio of one fraction (P or S) to the entire sample (P + S) and are depicted as mean ± SD of three biological replicates (*n* = 3).

As there is evidence that other α-defensins form aggregates with TcdB, this was next investigated for TcdB and α-def-6 in more detail. First, fluorescently labeled TcdB (TcdB-DL_488_) was incubated with α-def-6, and the formation of the aggregates was observed over time with a fluorescence microscope in the absence of cells. The presence of α-def-6 resulted in a very fast and distinct formation of large toxin-defensin complexes (Figure 2A, upper panel). However, in the absence of α-def-6, no formation of aggregates was observed (Figure 2A, lower panel).

Next, it was analyzed whether this aggregate formation also occurs in the presence of cells. Thus, Vero cells were incubated with TcdB-DL_488_ in the absence or presence of α-def-6. Afterwards, the cells were washed, fixed and stained with SiR-actin to visualize the actin cytoskeleton and to display the whole cell morphology. While the cells treated with TcdB-DL_488_ in the absence of α-def-6 showed clear and distinct rounding, the cells treated with TcdB-DL_488_ and α-def-6 showed no morphological changes. Remarkably, the toxin-defensins-aggregates were also visible in this setting, indicating that the toxin is still present, but no longer able to intoxicate cells (Figure 2B). Finally, aggregate formation was confirmed in a Western-blot-based precipitation assay. Here, incubation of TcdB with α-def-6 led to the shift of TcdB from the supernatant to the pellet fraction, indicating aggregate formation. Changing the incubation temperature from 37 °C to 4 °C led to the same results, hinting at a potential temperature-independent process (Figure 2C).

### 2.3. Vero Cells Are Protected from Intoxication with TcdB by α-def-6 in a Time- and Concentration-Dependent Manner

**Figure 3:**
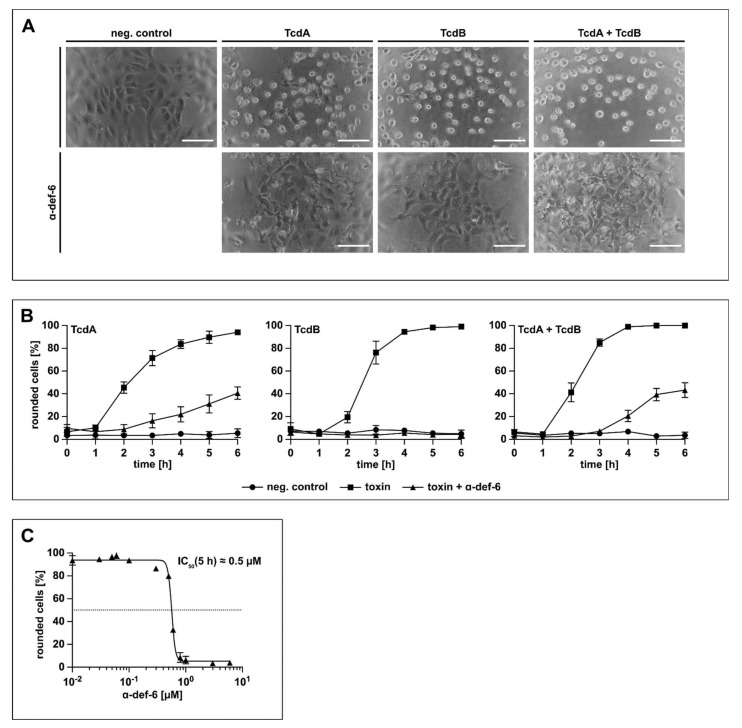
α-def-6 protects Vero cells from intoxication with TcdA, TcdB and the combination of both toxins. (**A**) Vero cells were intoxicated with TcdA (20 pM), TcdB (10 pM) or the combination of both toxins in the presence or absence of α-def-6 (6 µM). For control, the cells were left untreated. Representative images after 6 h incubation time are shown (*n* = 3). Scale bars correspond to 100 µm. (**B**) Vero cells were treated as in (**A**). The amount of rounded, i.e., intoxicated cells was determined hourly. Values are given as mean ± SD of three technical replicates. Three biological replicates exhibited comparable intoxication and inhibition (*n* = 3). (**C**) The concentration-dependent inhibition of TcdB (10 pM) was analyzed by incubating Vero cells with a concentration series of α-def-6 (ranging from 0.01 µM to 6 µM). After 5 h, images were taken, and the percentage of rounded cells was determined. Depicted is the mean ± SD of three technical replicates. Nonlinear fit was applied with GraphPad Prism via log(inhibitor) vs. response (variable slope, four parameters). Under this defined condition, an estimated IC_50_ value of about 0.5 µM was measured. The value was derived from three biological replicates, each with three technical replicates (*n* = 3).

Prompted by the finding that the α-def-6-mediated aggregation of TcdB neutralizes the toxin and protects cells from intoxication by TcdB, it was analyzed whether this defensin also neutralizes TcdA and the medically more relevant combination of both TcdA and TcdB. To this end, Vero cells were treated with TcdA, TcdB or TcdA + TcdB in the presence or absence of α-def-6. Vero cells are highly susceptible towards TcdA and TcdB and respond with clear morphological changes after intoxication (i.e., rounding of cells), which is a well-established, specific and sensitive endpoint to monitor the biological activities of TcdA and TcdB. The toxins and α-def-6 were added to the cells simultaneously without pre-incubation. As shown in Figure 3A, α-def-6 inhibited the single toxins TcdA and TcdB as well as the combination of both toxins in this approach. Moreover, the quantitative analysis of toxin-induced changes in cell morphology revealed a clear inhibition of TcdA, TcdB and TcdA + TcdB over time. Even after a comparatively long incubation period of 6 h, an almost complete protection against TcdB and a clear protection of the cells against TcdA and TcdA + TcdB could be observed (Figure 3B).

Exemplarily for TcdB, a clear concentration-dependent reduction of the amount of rounded cells at the defined time point of 5 h was observed when α-def-6 concentrations between 0.01 µM and 6 µM were applied. The result revealed an estimated half-maximal effective inhibitory concentration of approximately 0.5 µM of α-def-6 monitored under this particular experimental condition (Figure 3C).

### 2.4. Intracellular Rac1 In Vero Cells Is Protected from Toxin-Mediated Glucosylation in the Presence of α-def-6

**Figure 4:**
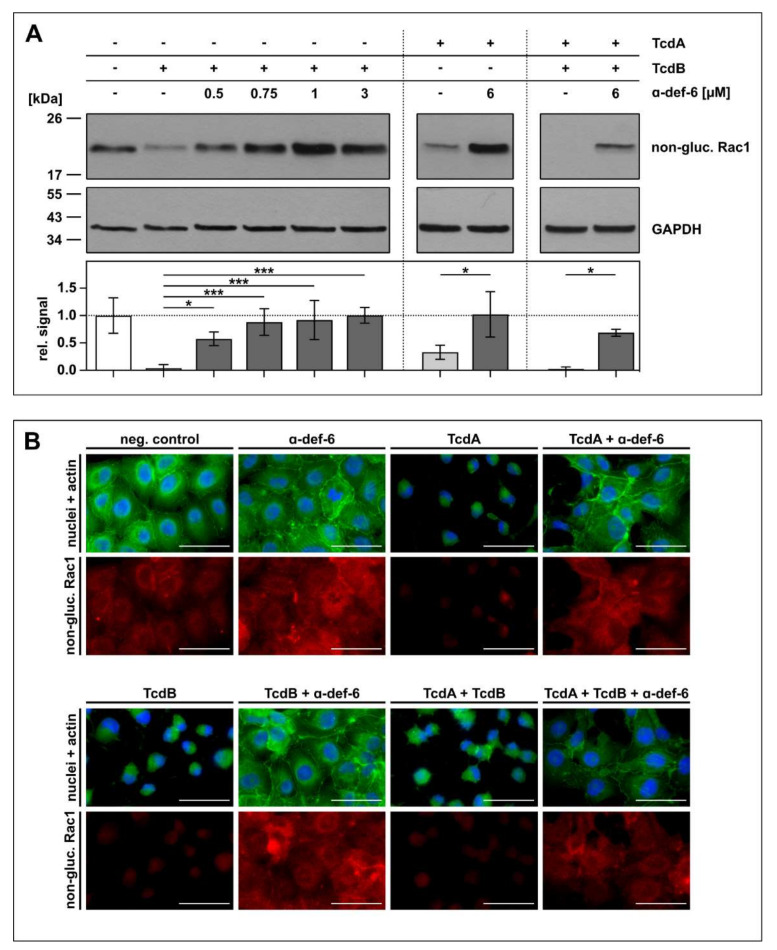
α-def-6 inhibits intracellular Rac1 glucosylation by TcdA, TcdB and the combination of both toxins. (**A**) Vero cells were treated with TcdA (20 pM), TcdB (10 pM) or the combination of both toxins with or without the addition of α-def-6 (concentrations as indicated) for 5 h (TcdB) or 4 h (TcdA and TcdA + TcdB), respectively. Then, the cells were harvested, lysed and subjected to SDS-PAGE followed by Western blotting. Non-glucosylated Rac1 was detected and signals were normalized to GAPDH as loading control. Representative Western blots are depicted. Relative signal intensities are given as mean ± SD of at least three biological replicates (*n* ≥ 3). Significance was tested with one-way ANOVA combined with Dunnett’s multiple comparison test (* *p* < 0.05, *** *p* < 0.001). (**B**) Vero cells were treated as in (**A**). α-def-6 (6 µM) was used alone for control. After 4 h, cells were fixed and permeabilized. Non-glucosylated Rac1 (red) was stained with an anti-Rac1-antibody in combination with a secondary Alexa Fluor_633_-conjugated antibody. Actin (green) was stained with phalloidin-FITC and nuclei (blue) via Hoechst33342. Representative images are shown (*n* = 2). Scale bars correspond to 50 µm.

To confirm the protective effect of α-def-6 against TcdA and TcdB by an alternative endpoint, the glucosylation status of intracellular Rac1, a substrate of TcdA and TcdB, was determined. Again, Vero cells were challenged with TcdA, TcdB and the combination of both toxins in the absence or presence of α-def-6. For control, the cells were left untreated. After defined time points, intoxication was stopped, the cells were lysed and subjected to SDS-PAGE and subsequent Western blot analysis for detection of non-glucosylated Rac1. Here, an antibody was used that specifically recognizes unmodified, i.e., non-glucosylated Rac1. Both toxins, TcdA and TcdB, glucosylate Rac1 in living cells, which results in a reduced signal in the Western blot analysis. On the protein level, clearly less Rac1 was glucosylated by the toxins in the presence of α-def-6. Moreover, also for the combination of both toxins, α-def-6 diminished the toxin-mediated Rac1-modification (Figure 4A).

Via immunofluorescence microscopy, the results obtained by Western blotting were confirmed. In this approach, less Rac1 was glucosylated in the presence of α-def-6 in intact cells displaying their native morphology and overall architecture (Figure 4B).

### 2.5. CaCo-2 Cells Are Protected from the Cytotoxic Effect of TcdA and TcdB in the Presence of α-def-6

**Figure 5:**
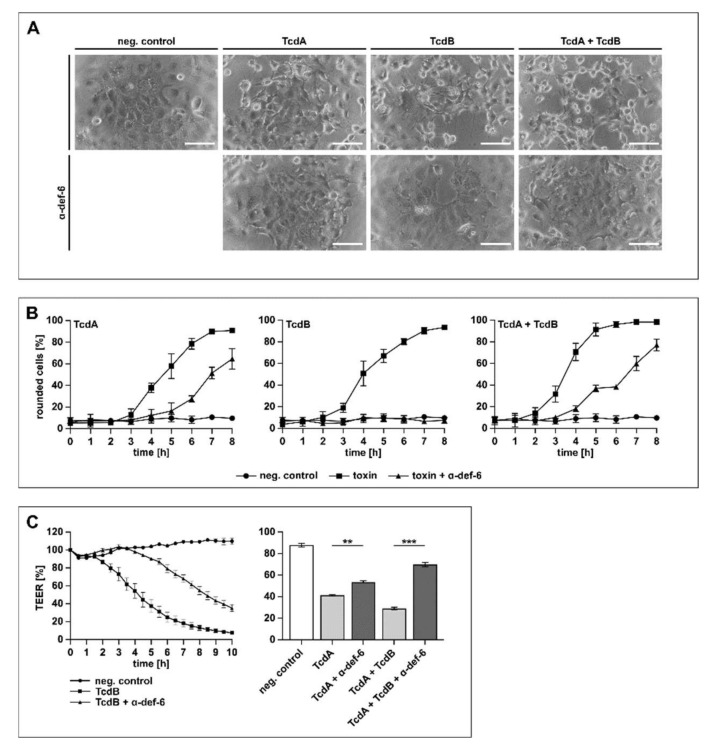
α-def-6 protects human CaCo-2 cells from intoxication with TcdA, TcdB and their combination. (**A**) CaCo-2 cells were treated with TcdA (500 pM), TcdB (50 pM) or the combination of both toxins in the presence and absence of α-def-6 (6 µM). For control, the cells were left untreated. Representative images after 6 h of incubation time are shown. Scale bars correspond to 100 µm. (**B**) CaCo-2 cells were treated as in (**A**). Images were taken each hour, and the amount of rounded cells over time was determined. Values are given as mean ± SD of three technical replicates. Three biological replicates showed comparable intoxication and inhibition (*n* = 3). (**C**) Transepithelial electrical resistance (TEER) was measured across a CaCo-2 monolayer. Cells were seeded in cell culture inserts (for TcdB: Millicell; for TcdA and TcdA + TcdB: Brand), and the toxins and α-def-6 (6 µM) were added apically. For TcdB (100 pM), a time-dependent protection of the CaCo-2 monolayer in the presence of α-def-6 is displayed (left panel). For TcdA (200 pM) and TcdA + TcdB, data after 2 h are shown (right panel). Values are given as mean ± SD. Significance was determined using a one-way ANOVA combined with a Dunnett’s multiple comparison test (** *p* < 0.01, *** *p* < 0.001). Three biological replicates showed comparable results (*n* = 3).

In the next set of experiments, the physiologically more relevant human colon carcinoma cell line (CaCo-2) was tested. The cells were treated with TcdA, TcdB and TcdA + TcdB in the presence or absence of α-def-6. Due to the fact that TcdA is significantly less effective than TcdB on cultured cells [30], different toxin concentrations were used to achieve a comparable intoxication over time. The toxin-induced morphological changes were quantified by counting rounded cells to allow for a graphical presentation of the intoxication and the inhibition. As shown in Figure 5A, the cells displayed obvious morphological changes after toxin treatment, while the presence of α-def-6 clearly diminished the amount of rounded cells.

When treated with TcdA, TcdB or TcdA + TcdB in the presence of α-def-6, a reduced amount of rounded CaCo-2 cells was visible for at least 6 h, indicating a time-dependent inhibition of the single toxins and the combination (Figure 5B). Finally, the protection of the transepithelial electrical resistance (TEER) of a CaCo-2 monolayer was investigated in more detail. This tight CaCo-2 cell monolayer mimics the situation of an epithelium in vitro. The toxin-mediated decrease in the epithelial integrity of the monolayer and the effect of α-def-6 was recorded over time (Figure 5C). The results indicated a clear delay in TEER-reduction in the presence of α-def-6 for all toxins tested. Illustrative for TcdB, a time-dependent inhibition of the cytotoxic effects on the monolayer could be observed in the presence of α-def-6 (Figure 5C, left panel). For TcdA and the combination of both toxins, protective effects after 2 h are displayed (Figure 5C, right panel).

### 2.6. α-def-6 Reduces the Cytotoxic Effects of TcdB in an In Vivo Zebrafish Embryo System

**Figure 6:**
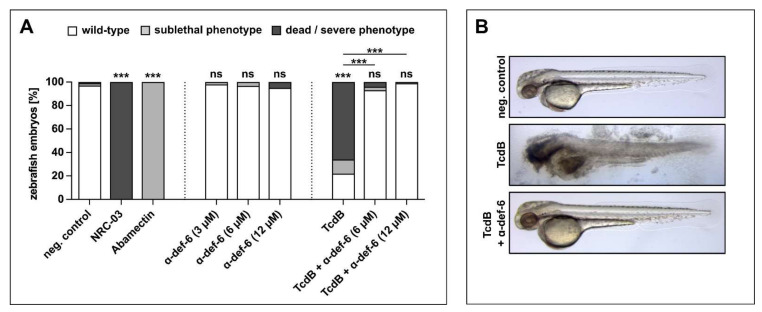
α-def-6 rescues zebrafish embryos from severe TcdB-induced damage and shows no self-toxicity. (**A**) Zebrafish embryos were incubated with TcdB (25 nM), α-def-6 or both. As negative control, embryos were subjected to the respective volume of solvent (PBS). NRC-03 (6 µM) served as a positive control for cytotoxicity, while Abamectin (3.125 µM) causes neurotoxicity. After 24 h of incubation, embryos (48 h post fertilization) were visually scored and categorized according to Supplementary Figure S1E. Embryos that displayed many necrotic cells (category Nec2), strong tissue damage (L3) or complete lysis (L4) and thus were dead at the time of analysis are plotted as “dead/severe phenotype”. Sublethal phenotypes comprise weaker cytotoxicity (limited necrosis or lysis), developmental toxicity (malformations or developmental delay), cardiotoxicity (impaired circulation or heart edema) and neurotoxicity (reduced escape movement). Data from three biological replicates are shown (two for α-def-6 only) (*n* = 90 embryos each, except for 60 for α-def-6 only and 75 for TcdB + α-def-6). Significance was tested using a Chi-Square test (ns = not significant, *p* > 0.05, *** *p* < 0.001). (**B**) Illustrative images displaying the typical wild-type appearance of neg. control- and rescue-embryos (TcdB + α-def-6 (12 µM)) and the strong tissue damage (L3) phenotype caused by TcdB (25 nM).

The findings found so far were finally verified in vivo using zebrafish embryos, a widely used model for investigating the effects of TcdB and therapeutics [31,32]. Embryos were exposed for 24 h starting at 24 h post fertilization, when most organ systems have already developed and are functional. The transparency of the embryos allowed for the evaluation not only of mortality but also of sublethal cytotoxicity (necrosis, lysis), developmental toxicity (developmental delay, malformations) or toxicity affecting specific organ systems, in particular cardiotoxicity (heart edema, reduced circulation) and neurotoxicity (reduced touch escape response) (Supplementary Figure S1). The pleurocidin antimicrobial peptide NRC-03 and the neurotoxin Abamectin were used as positive controls for cytotoxicity and neurotoxicity, respectively. First, different concentrations of α-def-6 alone were tested in this system. α-def-6 in concentrations that demonstrated anti-toxin activity in the previous experiments did not show any toxicity (Figure 6A), which was in line with the cell culture findings (Supplementary Figure S2).

Incubation of zebrafish embryos with TcdB caused death and severe cytotoxicity (lysis and necrosis) in >60% of embryos (Figure 6A, Supplementary Figure S1). Some of the less severely affected embryos also displayed sublethal cardiotoxicity (Figure 6A, Supplementary Figure S1). Remarkably, co-treatment with α-def-6 at 6 and 12 µM very efficiently reversed both the cytotoxic and cardiotoxic effects of TcdB (Figure 6A, B, Supplementary Figure S1).

Taken together, we identified the human antimicrobial peptide α-def-6 as a novel inhibitor of the clostridial glucosylating toxins TcdA and TcdB. The underlying mechanism is most likely based on the rapid entrapment of the toxins, leading to the formation of large toxin-defensin aggregates, but without affecting the glucosyltransferase activity of the toxins, which is in contrast to other human α-defensins. Noteworthy, the used α-def-6 concentrations as well as the resulting toxin-defensin-aggregates did not show any in vivo toxicity, making the defensin a favorable and novel candidate in the context of CDAD.

## 3. Discussion

*C. difficile*, the causative agent of CDIs, is one of the leading causes of nosocomial infections, which are associated with increasing costs in the health-care sector and high hospital admissions [33]. The major virulence factors in CDI pathogenesis are the large clostridial glucosylating toxins TcdA and TcdB. The presence of the toxins has been directly implicated in the emergence of the typical clinical symptoms mentioned above. *C. difficile* is a bacterium whose spores are transmitted through the fecal-oral route. Once in the gut, the spores germinate and express and secrete the toxins TcdA and TcdB, which bind to their specific host cell receptors, causing the typical clinical symptoms [34].

The results obtained in the present study confirm and extend earlier findings which showed that human α-defensins neutralize bacterial protein toxins [24,25,35,36]. We demonstrated that human α-def-6 specifically and directly binds to TcdB and induces the rapid formation of toxin aggregates, which likely entrap the toxin. The administration of α-def-6 protected human cells from TcdB, but also from the closely related TcdA and the clinically more relevant combination of both TcdA and TcdB. Exemplarily, the protective effect could be confirmed for TcdB in vivo using the zebrafish embryo model. Defensins in general are an important part of the innate immune system and build the first-line defense against infectious pathogens such as fungi, bacteria and several viruses. They are small, cysteine-rich, cationic peptides and exhibit a three-stranded β-sheet core structure, which is maintained and stabilized by three intramolecular disulfide bridges [37,38]. These structural elements make up their protease resistance and are the reason for the classification into α- and β-defensins [39]. Until today, six different human α-defensins were described, which can be further subdivided into human neutrophil peptides 1 to 4 (HNP 1–4) and human enteric defensins 5 and 6 (HD 5–6) [40,41]. HNPs are predominantly found in human neutrophils, whereas α-def-5 and -6 are produced and secreted by Paneth cells in the small intestine [42]. Paneth cells are located at the bases of the crypts of Lieberkühn [43,44] and release a package of antimicrobial peptides and other proteins into the enteric lumen. Especially, α-def-5 and -6 are known to influence the composition and constitution of the gut microbiota [45]. Most α-defensins benefit from their unique ability to destroy and disrupt bacterial cell membranes. They contain several conserved structures, such as the intra-molecular disulfide bridges, but differ in their amino acid sequences, which can lead to different biological functionalities. The comparison between α-def-5 and α-ef-6 is especially thrilling in this context. Both are highly similar and co-expressed in the same cells, but α-def-5 displays significant biocidal activity against invading pathogens, whereas α-def-6 possesses a surprisingly low antimicrobial activity [46]. Its non-canonical crystal structure is clearly different from the structures described for HNPs and α-def-5 [47]. It can assemble into an elongated quaternary super-structure arrangement [41] and is capable of self-oligomerizing into so-called nanonets [27,48,49]. This self-assembly process aims to protect the particularly vulnerable stem cells in the intestinal crypts of Lieberkühn from invading threats. The resulting α-def-6 oligomers entrap the bacteria in the intestinal lumen, preventing damage to the gut epithelium and the dissemination of the pathogens into other organs [48]. Interestingly, this behavior is also known from neutrophils, which form extracellular traps in whose net-like structures microbes become entrapped and killed [50]. Among the defensins, however, this behavior is unique to α-def-6.

There is increasing evidence that α-def-6 represents an outlier among the α-defensins, which is also noticeable in the context of bacterial toxins. Anti-toxin activities have already been described for several α-defensins. Kudryashova and colleagues showed in notable studies that putatively non-related bacterial toxins are affected by several α-defensins due to their thermodynamic instability, which is imperative for pore formation and finally for the translocation of the toxins across the endosomal membrane into the cytosol. For HNP1, but also to a lesser extent for α-def-5, it has been shown that their direct interaction with bacterial protein toxins leads to the destabilization of secondary and tertiary structures and thereby to a local unfolding of the toxins [51]. Interestingly, this anti-chaperone ability of several α-defensins is also thought to be responsible for the inactivation of viral proteins, including SARS-CoV-2 [52,53,54]. Again, it is worth to point out that α-def-6 cannot prevent the infection of cells with SARS-CoV-2 [55].

Also, the inhibition of bacterial protein toxins has yet to be observed for α-def-6. Thus, we have focused in the present study on the antimicrobial peptide α-def-6 and found that this defensin, despite its distinctly different properties compared to the other α-defensins, also protected cells from TcdA and TcdB. The proposed underlying molecular mechanism is to some extent comparable to that of the other α-defensins. The presence of α-def-6 leads to the rapid formation of toxin-defensin aggregates. This is in line with previously published results for HNP1 and α-def-5 [24,25,26] and occurs within a few seconds to minutes. This effect was independent of the presence of cells and also occurred at low temperature, suggesting a direct interaction between toxins and defensins. Nevertheless, the presence of cells did not hinder the aggregate formation, indicating that cellular factors such as proteases do not prevent or revert this process.

It is worth noting that there are also differences regarding the toxin neutralizing activity between α-def-5 and -6. α-def-5 directly inhibits the glucosyltransferase and glucosylhydrolase activity of TcdB [24], which was confirmed in the present study. However, no inhibition of the glucosyltransferase activity of TcdB was observed after incubation, with α-def-6, even at very high concentrations. This once again underlines the special character of α-def-6. Moreover, the results obtained in this study revealed that α-def-6 had no effect on the auto-proteolytic processing of TcdB.

Finally, the protective effects of α-def-6 against TcdB were confirmed in vivo using the zebrafish embryo model. Here, we could observe the previously shown toxicity of TcdB to the cardiovascular system [56], but also found more dramatic severe cytotoxic effects. Intriguingly, both were prevented in the presence of α-def-6. It should also be mentioned that the defensin alone had no adverse effects on cells or zebrafish embryos.

In the context of CDIs, which are associated with a dysfunctional intestinal flora, α-def-6 might be a potent inhibitor of the *C. difficile* toxins TcdA and TcdB. Given its endogenous origin within the human small intestine, α-def-6 might represent an interesting candidate in the search for novel therapeutic options against CDI locally administered in the colon.

## 4. Materials and Methods

### 4.1. Materials

The native toxins TcdA and TcdB from *C. difficile* VPI 10463 were expressed and purified as described earlier [57]. β-defensin-1, α-defensin-5 and α-defensin-6 were purchased from Pepta Nova (Sandhausen, Germany). Castanospermine was ordered from Santa Cruz Biotechnology, Inc. (Dallas, TX, USA) and N-Ethylmaleimide from Sigma Aldrich by Merck KGaA (Darmstadt, Germany). Recombinant Rac1 was expressed and used as a recombinant GST-tagged protein, as previously described [58].

### 4.2. Methods

#### 4.2.1. Cell Culture, Cytotoxicity and Cell Viability Assays

Cells were grown in their respective media under humidified conditions at 37 °C and 5% CO_2_. Vero cells (African green monkey kidney cells; DSMZ, Braunschweig, Germany) were maintained in Gibco minimum essential medium (MEM; Thermo Fisher Scientific, Waltham, MA, USA) supplemented with 10% fetal calf serum (FCS; Thermo Fisher Scientific, Waltham, MA, USA), 1 mM sodium pyruvate (Thermo Fisher Scientific, Waltham, MA, USA), 2 mM L-glutamine (PAN-Biotech, Aidenbach, Germany), 0.1 mM non-essential amino acids (Thermo Fisher Scientific, Waltham, MA, USA) and 100 U/mL penicillin and 100 g/mL streptomycin (both Thermo Fisher Scientific, Waltham, MA, USA). CaCo-2 cells (human epithelial colorectal adenocarcinoma cells, HTB-37; ATCC, Manassas, VA, USA) were cultured in Gibco Dulbecco’s Modified Eagle Medium (DMEM; Thermo Fisher Scientific, Waltham, MA, USA) plus 10% FCS, 1 mM sodium pyruvate, 0.1 mM non-essential amino acids and 100 U/mL penicillin and 100 g/mL streptomycin.

For the cytotoxicity assays, cells were grown in a multi-well-format until reaching the desired confluency and subsequently treated with TcdA, TcdB or the combination of both toxins (toxin dilutions in FCS-containing medium, concentrations as indicated) in FCS-free medium in the absence or presence of inhibitors. Cell rounding was visualized by light microscopy using a Leica DMi1 microscope connected to a Leica MC170 HD camera (both Leica Microsystems GmbH, Wetzlar, Germany). Images were taken hourly. Intoxication was quantified as the ratio of rounded cells to the total cell number.

For the cell viability assays, Vero cells were seeded in a 96-well plate and subjected to α-def-6 (6 µM), DMSO (30% (*v*/*v*)) or H_2_O in FCS-containing MEM for the indicated time frames. Cell viability was analyzed by addition of the CellTiter 96 AQ_ueous_ One Solution Cell Proliferation Assay (Promega Corporation, Madison, WI, USA) and subsequent incubation for 1 h at 37 °C. An absorbance measurement at 490 nm was performed using the TriStar^2^ LB942 multimode reader (Berthold Technologies GmbH & Co. KG, Bad Wildbad, Germany).

#### 4.2.2. Biotinylation of TcdB

TcdB was biotinylated via EZ-Link^TM^ Sulfo-NHS-Biotin (Thermo Fisher Scientific, Waltham, MA, USA) according to the manufacturer’s protocol. Briefly, TcdB was incubated with Sulfo-NHS-Biotin for 30 min at room temperature (RT). To remove the excess non-reacted biotin, TcdB was rebuffered with a Zeba^TM^ Spin Desalting Column (Thermo Fisher Scientific, Waltham, MA, USA). The successful biotinylation was verified via Western blotting and detection with peroxidase-conjugated streptavidin (1:2500, F. Hoffmann-La Roche AG, Basel, Switzerland). The biotinylated TcdB showed unimpaired activity in a cytotoxicity assay.

#### 4.2.3. Surface Plasmon Resonance Measurements

The binding of α-def-6 to biotinylated TcdB was analyzed via surface plasmon resonance spectroscopy (SPR) with a Reichert SPR7500DC SPR spectrometer (Reichert Technologies, Buffalo, NY, USA). All analytes were dialyzed in running buffer (10 mM HEPES, 150 mM NaCl, 50 µM EDTA, 0.005% (*v*/*v*) Tween^®^20, pH 7.0) beforehand. Experiments were performed with a flow rate of 25 µL/min at 25 °C. A streptavidin sensor chip (SAD500m; XanTec bioanalytics GmbH, Duesseldorf, Germany) was conditioned and washed according to the manufacturer’s recommendation. Biotinylated TcdB was attached to the surface of one of the flow cells via streptavidin-biotin binding. In total, 2400 response units (RUs) were immobilized. All analytes were injected for 4 min followed by a dissociation phase with a running buffer flow for at least 30 min. For α-def-6, various concentrations were tested (0.1 µM, 1 µM, 3 µM, 6 µM). The highest concentration was injected twice to assess reproducibility. β-def-1 (6 µM) served as a control.

#### 4.2.4. In Vitro Autoprocessing of TcdB

The effects of α-def-6 on TcdB autoprocessing by its intrinsic cysteine protease activity were analyzed. TcdB (2 µg) was incubated in the absence or presence of α-def-6 (3 µM, 9 µM) for 1 h at 37 °C, buffered with 20 mM Tris-HCl and 150 mM NaCl at pH 7.4 in a final volume of 20 µL. Inositol hexakisphosphate (1 mM, Santa Cruz Biotechnology, Dallas, TX, USA) was added to the samples to induce autoprocessing. *N*-Ethylmaleimide (1 mM, Sigma Aldrich by Merck KGaA, Darmstadt, Germany) served as positive control for the inhibition of TcdB-autoprocessing. The reactions were stopped by addition of a Laemmli buffer and heat denaturation (95 °C). For the analysis, the samples were electrophoresed on 8% SDS polyacrylamide gels, and proteins were visualized by Coomassie staining.

#### 4.2.5. In Vitro Glucosylation of Rac1 by TcdB

To evaluate the potential effects of α-def-6 on TcdB glucosyltransferase activity, 40 µg of CaCo-2 cell lysate were used as a source of Rac1 and incubated with TcdB (50 ng) in glucosylation buffer (50 mM HEPES, 100 mM KCl, 2 mM MgCl_2_, 1 mM MnCl_2_, 100 mg/L BSA, pH 7.5) in a final volume of 20 µL. Two concentrations of α-def-6 (6 µM, 12 µM) were tested. Reactions were allowed to proceed for 2 h at 37 °C and stopped with the addition of a Laemmli buffer and heating for 10 min at 95 °C. The samples were loaded onto a 12.5% polyacrylamide gel and separated via SDS-PAGE followed by Western blotting. Unspecific binding sites were blocked with 5% (*w*/*v*) skim milk powder diluted in PBS with 0.1% (*v*/*v*) Tween^®^20 (PBS-T, pH 7.4) for 1 h at RT. The glucosylation status of Rac1 was assessed by probing with a mouse anti-Rac1-antibody (1:500, #610651, BD Biosciences, Franklin Lakes, NJ, USA) specifically directed against non-glucosylated Rac1 overnight at 4 °C. After washing, the membrane was incubated with a secondary horseradish-peroxidase (HRP)-conjugated goat-anti-mouse antibody (1:2500, #31430, Invitrogen by Thermo Fisher Scientific, Waltham, MA, USA) for 1 h at RT. Signals were detected using the ECL-system. GAPDH (mouse anti-GAPDH antibody, 1:2000, #sc-365062) followed by mouse IgG kappa binding protein-HRP (1:2500, #sc-516102; both Santa Cruz Biotechnology, Dallas, TX, USA) served as loading control. Densitometric analysis was performed using ImageJ (v1.53p, U.S. National Institutes of Health, Bethesda, MD, USA), and the signals were normalized to loading control.

#### 4.2.6. UDP-Glo^TM^ Glucosyltransferase Assay

UDP-Glo™ Glucosyltransferase assay with UDP-Glucose (Promega Corporation, Madison, WI, USA) was performed according to the manufacturer’s protocol. Briefly, TcdB (200 pM) was incubated in glucosylation buffer (50 mM HEPES, 100 mM KCl, 2 mM MgCl_2_, 1 mM MnCl_2_, 100 mg/L BSA, pH 7.5) with various concentrations of the respective inhibitors in a final volume of 40 µL. 5 µM recombinant Rac1 was included in the samples as a substrate. Reactions were started by addition of 100 µM UDP-glucose and allowed to proceed for 1 h at 37 °C. Castanospermine (10 mM, Santa Cruz Biotechnology, Dallas, TX, USA) served as control for inhibition of glucosyltransferase activity of TcdB. To stop the reaction, three times 10 µL of each sample were transferred to a white half area 96 well microplate (Greiner Bio-One International GmbH, Kremsmünster, Austria) combined with 10 µL of UDP Detection Reagent and mixed at 1000 rpm for 30 s on a plate shaker (Titramax 1000, Heidolph Instruments GmbH & Co. KG, Schwabach, Germany). Luminescence was recorded after 15–60 min via a Tecan infinite M1000 Pro plate reader (Tecan, Männdorf, Switzerland) with an integration time of 750 ms.

#### 4.2.7. Fluorochrome Labeling of TcdB

TcdB was labeled using the DyLight^TM^ 488 NHS-Ester (Thermo Fisher Scientific, Waltham, MA, USA) according to the manufacturer’s protocol. Simultaneously the same volume of PBS was exposed to DyLight^TM^ 488 NHS-Ester to serve as a negative control in experiments. To remove free fluorescence dye, the samples were rebuffered by passing through Zeba^TM^ Spin Desalting Columns via centrifugation. The DyLight^TM^ 488 labeled TcdB (TcdB-DL_488_) showed unchanged activity in a cytotoxicity assay.

#### 4.2.8. Aggregation of TcdB-DL_488_ over Time in the Absence of Cultured Cells

α-def-6 (6 µM) was added to TcdB-DL_488_ (30 nM) in PBS in an 8-well or 18-well µ-slide (ibidi GmbH, Gräfelfing, Germany), and image acquisition was started immediately with an iMIC digital microscope using the Live Acquisition 2.6 software (both FEI Munich GmbH by Thermo Fisher Scientific, Waltham, MA, USA). Aggregation was allowed to proceed for 30 min at 37 °C. Images were taken every second for the first 5 min and every 10 s for additional 25 min. Images were processed using ImageJ.

#### 4.2.9. Aggregation of TcdB-DL_488_ in the Presence of Cultured Cells

Vero cells were seeded in an 8-well or 18-well µ-slide (ibidi GmbH, Gräfelfing, Germany) and grown for one day. The cells were treated with TcdB-DL_488_ (22 nM) in the absence or presence of α-def-6 (6 µM) for 0.5 h at 37 °C. As a control, the cells were subjected to the same volume of DyLight^TM^488-treated PBS. The cells were washed with PBS, fixed with 4% (*w*/*v*) paraformaldehyde for 20 min at RT and permeabilized via incubation with 0.4% (*v*/*v*) Triton-X-100 followed by 100 mM glycine for 2 min at RT. To block unspecific binding sites, cells were treated with 5% (*w*/*v*) skim milk powder in PBS-T for 30 min at 37 °C. Next, actin was stained via incubation with SiR-actin (1:1000, SC001, Spirochrome AG, Stein am Rhein, Switzerland) for 1 h at 37 °C, followed by nuclei staining with Hoechst33342 (1:5000, Thermo Fisher Scientific, Waltham, MA, USA) for 5 min at 37 °C. The cells were washed with PBS, and images were taken with an iMIC digital microscope using the Live Acquisition 2.6 software. Images were processed using ImageJ.

#### 4.2.10. Precipitation Assay with TcdB

The TcdB stock was centrifuged for 20 min at 14,000 rpm and 4 °C to remove any preformed aggregates. TcdB (50 ng) was incubated in the absence or presence of α-def-6 (6 µM) for 30 min at 37 °C in a final volume of 35 µL PBS. The samples were centrifuged as before to segregate possible aggregates and subsequently separated into a supernatant (30 µL) and pellet fraction (5 µL). The pellet fraction was resuspended with 25 µL PBS to a final volume of 30 µL. Each fraction (30 µL) was then subjected to SDS-PAGE (8% polyacrylamide gel) and Western blotting. TcdB was detected using an anti-TcdB-antibody (1:1000, #ab270452, Abcam, Cambridge, UK) followed by an HRP-conjugated mouse anti-rabbit antibody (1:2500; #sc-2357; Santa Cruz Biotechnology, Dallas, TX, USA).

#### 4.2.11. Intracellular Rac1 Status after Treatment with TcdA or TcdB

Vero cells (80,000/well) were grown in a 24-well plate for one day and intoxicated with TcdA (20 pM) and/or TcdB (10 pM) in the absence or presence of α-def-6 (concentrations as indicated). Intoxication was allowed to proceed for 4–5 h. The cells were washed, mechanically harvested in PBS plus 1× cOmplete Protease Inhibitor (F. Hoffmann-La Roche AG, Basel, Switzerland) and lysed via freezing. Samples were loaded onto a 12.5% polyacrylamide gel and separated via SDS-PAGE followed by Western blotting. Non-glucosylated Rac1 and GAPDH were detected, as described above.

#### 4.2.12. Immunofluorescence Microscopy

Vero cells (40,000/well) were seeded in an 8-well µ-slide (ibidi GmbH, Gräfelfing, Germany) and grown for one day. The medium was removed, and the cells were treated with either TcdA (20 pM), TcdB (10 pM) or the combination of both toxins in the absence or presence of α-def-6 (6 µM). Additionally, the cells were incubated with α-def-6 (6 µM) alone. After 4 h incubation time, the cells were washed with PBS and fixed with 4% (*w*/*v*) paraformaldehyde for 20 min at RT. Subsequently, the cells were permeabilized with 0.4% (*v*/*v*) Triton X-100 in PBS for 5 min followed by 100 mM glycine in PBS for 2 min at RT. To block unspecific binding sites, the cells were treated with 5% (*w*/*v*) skim milk powder in PBS-T for 30 min at 37 °C. Next, the cells were immunostained with a mouse anti-Rac1-antibody, specifically binding to non-glucosylated Rac1 for 30 min at 37 °C. After washing with PBS, the cells were probed with a fluorescent labeled secondary antibody (goat anti-mouse Alexa Fluor 633, 1:750, #A-21053, Invitrogen from Thermo Fisher Scientific, Waltham, MA, USA) for 30 min at 37 °C. Simultaneously, actin was stained with phalloidin-FITC (1:100, #P5282, Sigma Aldrich from Merck KGaA, Darmstadt, Germany). The cells were washed and nuclei-stained via incubation with Hoechst33342 (1:5000, Thermo Fisher Scientific, Waltham, MA, USA) for 5 min at 37 °C. Immunofluorescence microscopy images were taken with the iMic digital microscope using the Live Acquisition 2.6 software. The images were processed using ImageJ.

#### 4.2.13. Transepithelial Electrical Resistance (TEER) Measurements

Caco-2-cells (125,000/well) were seeded in 24-well cell culture inserts with a 0.4 µm pore size (for TcdB: Millicell Hanging Cell Culture Insert, Millipore by Merck KGaA, Darmstadt, Germany; for TcdA and TcdA + TcdB: BRAND inserts, BRAND GMBH + CO KG, Wertheim, Germany) and grown for four days. TcdA (200 pM) and/or TcdB (100 pM) as well as α-def-6 (6 µM) were added apically. H_2_O was included in the negative and positive controls as a solvent control for α-def-6. TEER was measured every 0.5 h with the EVOM^2^ apparatus connected to the STX2 electrode (both World Precision Instruments Inc., Sarasota, FL, USA). Raw resistance data were normalized to time point t = 0 h.

#### 4.2.14. Zebrafish Experiments

For in vivo toxicity studies in zebrafish (*Danio rerio*), wild-type embryos were dechorionated at 24 h post fertilization (hpf) using digestion with 1 mg/mL pronase (Sigma Aldrich from Merck KGaA, Darmstadt, Germany) in an E3 medium (83 μM NaCl, 2.8 μM KCl, 5.5 μM CaCl_2_, 5.5 μM MgSO_4_). In 96-well plates, three embryos per well were exposed for 24 h to 100 µL of E3 containing test substances (concentrations indicated in the figures). Three independent assays were performed with 10 wells per treatment each, except for treatments with α-def-6 alone, which were performed twice. The toxin solvent (PBS), diluted in E3, was used as negative control at the same amount as introduced by the toxin. As positive control for cytotoxicity, the pleurocidin antimicrobial peptide NRC-03 (GRRKRKWLRRIGKGVKIIGGAALDHL-NH2) was used at a concentration of 6 µM [59]. Abamectin at a concentration of 3.125 µM was used as positive control for neurotoxicity [60]. At 48 hpf (after 24 h of incubation), embryos were scored in a stereomicroscope for signs of cytotoxicity (lysis and/or necrosis, which is visible as loss of transparency), developmental toxicity (delay and/or malformations) or cardiotoxicity (heart edema and/or reduced or absent circulation). Each embryo was also touched with a needle, and reduced or absent touch responses (escape movements) were evaluated as signs of neurotoxicity if and only if no signs of cytotoxicity were present in the same embryo. The embryos were categorized within each of these toxicity categories into several classes of severity, as detailed in Supplementary Figure S1E. Severe lysis (class L4 and L3) and massive necrosis (Nec2) result in embryo death by the time of the analyses, while embryos displaying the other phenotypic classes were still alive. Severely affected embryos (L4, L3, Nec2) were not analyzed for other potential phenotypes and were excluded from the graphs presenting data on developmental toxicity, cardiotoxicity and neurotoxicity. A chi-Square test was used to calculate whether the distribution of embryos into toxicity classes differed significantly between treatment groups.

#### 4.2.15. Reproducibility of Experiments and Statistics

All experiments were performed independently at least twice. The number of biological replicates (*n*) for each experiment is given in the figure legends. Representative results are depicted in the figures. If not stated otherwise, a one-way ANOVA in combination with Dunnett’s multiple comparison test was performed for statistical analysis using GraphPad Prism Version 9 (GraphPad Software Inc., San Diego, CA, USA). The obtained *p* values are depicted as follows: ns = not significant *p* > 0.05, * *p* < 0.05, ** *p* < 0.01, *** *p* < 0.001, **** *p* < 0.0001.

## Data Availability

The original contributions presented in this study are included in the article/supplementary material. Further Inquiries can be directed to the corresponding authors.

## Supplementary Materials

The following supporting information can be downloaded at: https://www.mdpi.com/article/10.3390/ijms23094509/s1.
