# Supplementary material for: Human α-Defensin-6 Neutralizes Clostridioides difficile Toxins TcdA and TcdB by Direct Binding
Source: Int J Mol Sci. 2022 Apr 19;23(9):4509. doi: 10.3390/ijms23094509 (PMC9101188; doi:10.3390/ijms23094509)
Supplement: Supplementary file 1 [file ijms-23-04509-s001.zip › ijms-1679864-supplementary.pdf]

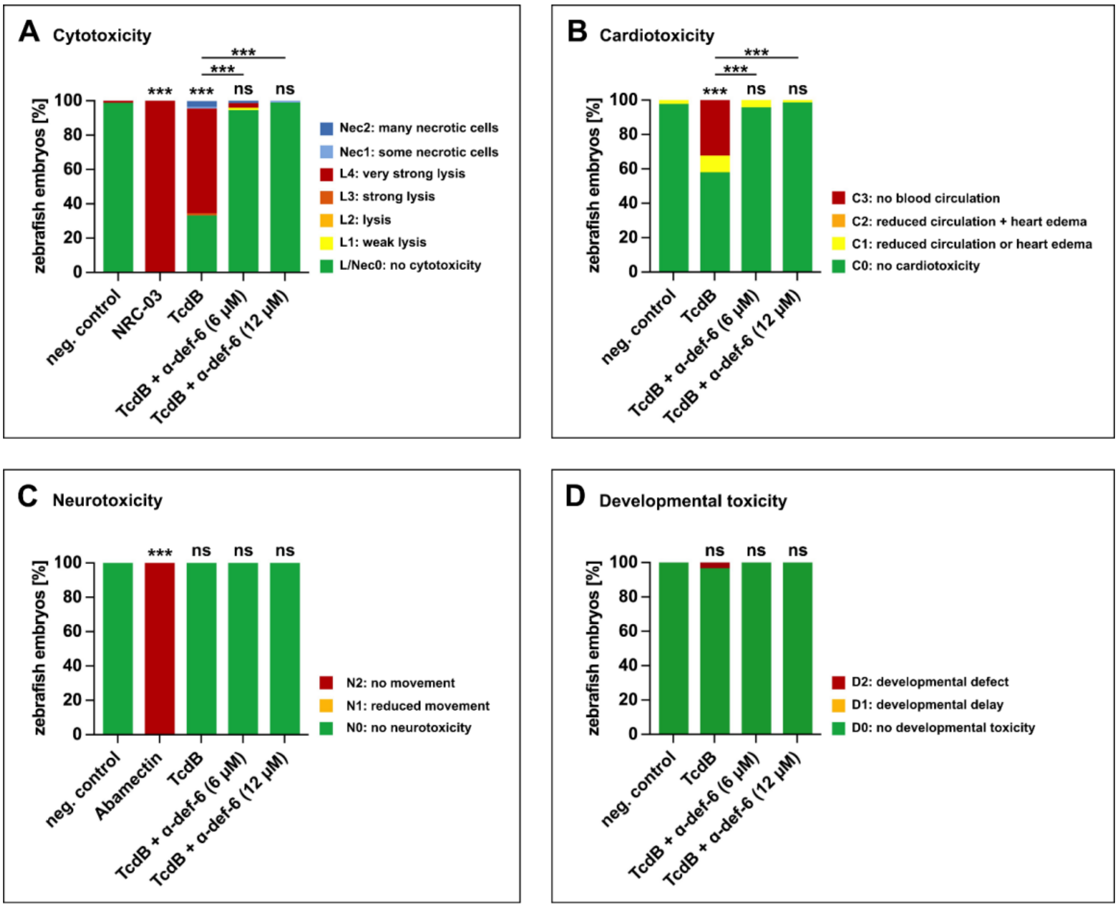

E

**Cytotoxicity**	
L1:	few lysed cells floating in medium; embryos look like wt
L2:	lysed cells in medium; embryos show some visible tissue damage
L3:	embryos show strong tissue damage
L4:	embryos are completely disintegrated
Nec1:	individual necrotic cells (darkened areas in brightfield)
Nec2:	many necrotic cells (darkened areas in brightfield)
**Developmental toxicity**	
D1:	developmental delay (slow development)
D2:	developmental defect (malformations)
**Cardiotoxicity**	
C1:	reduced circulation or heart edema
C2:	reduced circulation plus heart edema
C3:	no circulation with or without heart edema
**Neurotoxicity**	
N0:	normal movement in response to touch
N1:	reduced movement in response to touch
N2:	no movement in response to touch
**Overall toxicity (combination of above phenotypes)**	
wt:	wild type (no visible phenotype AND normal movement)
T1:	embryos that show a phenotype
T2:	severe damage so that other phenotypes cannot be assessed (L3, L4, Nec2)

Figure S1. TcdB causes cytotoxicity and cardiotoxicity in zebrafish embryos, which are both prevented by α-def-6. Embryos were incubated for 24 h with the indicated substances and scored for (**A**) cytotoxicity (lysis, necrosis), (**B**) cardiotoxicity (heart edema and/or reduced/absent circulation), (**C**) neurotoxicity (reduced or absent escape movements in response to touch) and (**D**)

developmental toxicity (developmental delay or malformations). As negative control, embryos were subjected to the respective volume of solvent (PBS). Embryos that showed severe phenotypes (L4: very strong lysis, L3: strong lysis, Nec2: many necrotic cells) and were dead at the time of analysis were excluded for the analyses of cardiotoxicity, developmental toxicity and neurotoxicity. Chi-Square test was used to test for significant differences of class distribution between the neg. control and the indicated columns, or between columns identified with lines. Data are from three biological replicates. n (cytotoxicity) = 90, 90, 90, 75, 74 embryos. n (cardiotoxicity, neurotoxicity, developmental toxicity) = 89, 31, 72, 74 embryos. (**E**) Criteria for scoring embryos into toxicity categories.

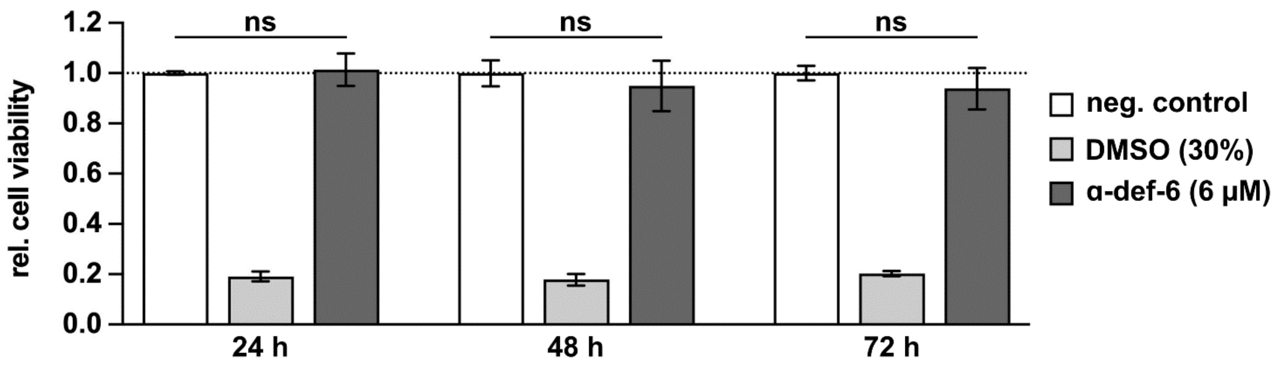

Figure S2. α-def-6 shows no effect on viability of cultured Vero cells. Vero cells were treated with α-def-6 (6 µM) for 24 h, 48 h and 72 h at 37 °C. Control cells were subjected to the respective volume of solvent (H_2O). 30% (*v*/*v*) DMSO served as a positive control for reduced cell viability. CellTiter 96 AQueous One Solution Cell Proliferation Assay was used to measure cell viability. Obtained absorbance values were normalized to the respective negative control and are shown as mean ± SD of three biological replicates each with three technical replicates (n = 3). Significance was tested with one-way ANOVA combined with Dunnett´s multiple comparison test (ns = not significant $p > 0.05$).
